# Circulating lipid profiles and post-prandial glucose and insulin in response to dietary macronutrient composition in lean and obese cats

**DOI:** 10.1093/jvimsj/aalag013

**Published:** 2026-02-17

**Authors:** Hannah Godfrey, Érico M Ribeiro, Shoshana Verton-Shaw, Anna Kate Shoveller, Darcia Kostiuk, Janelle Kelly, Jennifer Saunders Blades, Ron Johnson, Adronie Verbrugghe

**Affiliations:** Department of Biomedical Sciences, Ontario Veterinary College, University of Guelph, Guelph, Ontario, Canada; Department of Clinical Studies, Ontario Veterinary College, University of Guelph, Guelph, Ontario, Canada; Department of Clinical Studies, Ontario Veterinary College, University of Guelph, Guelph, Ontario, Canada; Department of Animal Biosciences, Ontario Agricultural College, University of Guelph, Guelph, Ontario, Canada; Champion Petfoods Holding Inc., Department of Research, Innovation and Product Development, Morinville, Alberta, Canada; Champion Petfoods Holding Inc., Department of Research, Innovation and Product Development, Morinville, Alberta, Canada; Champion Petfoods Holding Inc., Department of Research, Innovation and Product Development, Morinville, Alberta, Canada; Department of Biomedical Sciences, Ontario Veterinary College, University of Guelph, Guelph, Ontario, Canada; Department of Clinical Studies, Ontario Veterinary College, University of Guelph, Guelph, Ontario, Canada

**Keywords:** cholesterol, dietary carbohydrate, dietary fat, dietary protein, feline nutrition, insulin sensitivity, lipoproteins, whole blood glucose

## Abstract

**Background:**

Insulin response to a meal is crucial for metabolic health in cats, influencing the risk of metabolic disorders.

**Hypothesis/Objectives:**

Investigate dietary macronutrient compositions on fasted and post-prandial insulin and glucose responses, and lipid profiles, in lean and obese cats.

**Animals:**

Nine lean and 9 obese, male neutered colony cats.

**Methods:**

Cats were fed 3 extruded dry diets: low protein (LP: 28% protein, 40% fat, and 32% nitrogen-free extract [NFE]), low fat (LF: 40% protein, 30% fat, and 30% NFE), and low carbohydrate (LC: 36% protein, 41% fat, and 23% NFE) for 28 days using a 3 × 3 Latin square design. Fasted and post-prandial blood samples were collected to measure serum insulin and whole blood glucose concentrations, and fasted samples were analyzed for serum cholesterol, triacylglycerol (TAG), nonesterified fatty acid (NEFA), very low-density lipoprotein (VLDL), low-density lipoprotein cholesterol (LDL-c), and high-density lipoprotein cholesterol (HDL-c) concentrations at the end of each period.

**Results:**

No differences were found in serum insulin, glucose, cholesterol, NEFA, TAG, HDL-c, LDL-c, or VLDL concentrations between lean and obese cats (*P* > .05) suggesting dyslipidemia was not present in the obese cats. The LP diet resulted in lower post-prandial insulin concentrations compared with the LC and LF diets (*P* = .01) which was attributed to lower protein intake with the LP diet. As expected, the LF diet led to lower fasted serum cholesterol and LDL-c concentrations compared with the LP and LC diets (*P* < .001).

**Conclusions and clinical importance:**

These findings document the metabolic flexibility of cats and suggest that dietary macronutrient composition, particularly protein content, plays an important role in modulating insulin responses in adult, otherwise healthy, cats.

## Introduction

In humans and animals, obesity is a precursor to a cluster of metabolic changes (systemic inflammation, insulin resistance [IR], dyslipidemia, and hormonal imbalances) collectively referred to as the metabolic syndrome.^1–3^ Although in cats, the specific clinical definition is not standardized, this pattern of obesity and metabolic dysfunction is increasingly recognized and studied in feline medicine. Specifically, obesity in cats has a similar progression to type II diabetes (DM) as seen in humans.^2,4–7^ Impaired glucose tolerance is observed in prediabetic humans^4^ and IR in cats.^8–13^ In humans, it is hypothesized that chronic post-prandial hyperglycemia leads to higher demand for insulin from pancreatic β-cells, resulting in hyperinsulinemia and ultimately β-cell failure.^2,14,15^ Therefore, it is hypothesized that decreased demand on pancreatic β-cells by minimizing the post-prandial increase in blood glucose concentration may decrease the risk of developing DM in cats.

The dietary macronutrient distribution of the diet plays a role in modulating post-prandial insulin and glucose responses, and is a major focus for prevention and management of IR and DM. In humans, decreasing dietary carbohydrates is a common strategy.^2,4^ Similarly, in cats there is particular interest in the high-carbohydrate content (referring to nitrogen-free extract [NFE]) of commercially available cat foods compared with that of the typical prey species^16–19^ as the driver for the positive association of feeding extruded dry food with obesity onset and IR.^19,20^ Alternatively, a meta-analysis concluded that the NFE content as percentage of the metabolizable energy (%ME) of the diet in cats was not associated with changes in fasted glucose and insulin concentrations. Rather, dietary fat content may play a more important role in obesity and IR.^21^ The post-prandial insulin and glucose response was not addressed.

In healthy, adult cats, a previous study evaluated a novel approach to assess the effect of dietary macronutrient distributions on insulin sensitivity using intravenous glucose tolerance tests (IVGTTs) and formulating 3 test diets using a pair-wise isoenergetic decrease of each macronutrient to elucidate the separate effect of each energy source.^22^ Previously, we used this dietary approach and found that, similar to previous studies,^23–26^ cats were capable of adjusting to acute feeding (2 weeks) of variable macronutrient distributions as noted by the higher respiratory quotient after a low-fat (LF; protein 40%ME, fat 27%ME, NFE 27%ME) diet compared with the low-carbohydrate (LC; protein 35%ME, fat 40%ME, NFE 20%ME) diet.^27^ In addition, a LF (protein 40%ME, fat 30%ME, NFE 30%ME) diet resulted in higher lean soft tissue mass compared with LC (protein 36%ME, fat 41%ME, NFE 23%ME) and low-protein (LP; protein 28%ME, fat 40%ME, NFE 32%ME) diets^28^ following the same isoenergetic reduction approach.^22,27^ It is hypothesized that these changes after consumption of a LF diet are caused by a larger insulin response, resulting in more glucose flux into skeletal muscle for glucose oxidation and glycogen synthesis.^27–30^ We aimed to build upon these findings by formulating 3 diets that differed in macronutrient content using the same pairwise isoenergetic reduction approach^22^ to investigate the effect of dietary interventions on serum insulin and whole blood glucose responses in lean and obese cats, as well as changes in serum cholesterol and lipoprotein concentrations.

## Materials and methods

### Animals and housing

All experimental procedures were carried out in accordance with national and institutional guidelines for the care and use of animals in research and were approved by the University of Guelph Animal Care Committee (AUP #4865). Male neutered domestic shorthair cats (*n* = 18) with a mean age of 3.4 (0.28) years of age were group housed in a 7.0 m × 5.8 m free-living environment (Animal Biosciences Cat Colony, University of Guelph, Guelph, ON) on a 12-h light and dark cycle as previously described.^28^ Cats were classified using a 9-point body condition score (BCS) scale^31^ as lean (*n* = 9; BCS, 4/9 or 5/9) or obese (*n* = 9; BCS, > 8/9). Duration of obesity in the obese group was approximately 6 months before the start of the study. Mean and SD BW for lean and obese cats was 4.38 (0.25) kg and 5.80 (0.23) kg, respectively. All animals enrolled in the study were deemed healthy, except for an obese condition, based on results of physical examination, CBC, and serum biochemical profile.

### Dietary treatments and feeding

Cats were fed a commercially available extruded dry diet for adult maintenance (Orijen Original Cat, Champion Petfoods, Edmonton, AB, Canada) at an amount necessary to maintain body weight (BW) for 4 weeks before the study. The 3 experimental diets were formulated for adult maintenance^32^ using the same ingredients at variable amounts after an isoenergetic reduction for each macronutrient^22,27,28^ resulting in a pairwise change in macronutrient content for a LP (protein, 28%ME; fat, 40%ME; NFE, 32%ME), LF (protein, 40%ME; fat, 30%ME; NFE, 30%ME), and LC (protein, 36%ME; fat, 41%ME; NFE, 23%ME) diets. Compared with the LF and LP diet, the LC diet differed by isoenergetic substitution of carbohydrate for fat and carbohydrate for protein, respectively. Vitamin and mineral concentrations as well as physical structure of the kibble were formulated to be similar across diets. All 3 diets were evaluated for proximate analysis and total dietary fiber following the appropriate methods outlined by the Association of Official Analytical Chemists and American Oil Chemist Society, as previously described^28^ and summarized in Table S1. Metabolizable energy was calculated using modified Atwater factors^33,34^ and the NFE was estimated as the sum of crude protein (% as is), crude fat (% as is), crude fiber (% as is), and ash (% as is) subtracted from 100.^33^ Cats were offered food at an amount to maintain BW using historical colony data before the start of the study for 1 h per day (08:00 h) in individual cages. Food intake was measured daily and water was offered ad libitum.

### Experimental design

Cats were assigned to one of 3 groups (*n* = 6 per group) balanced for BW to receive each test diet for 24 days in a random order following a 3 × 3 Latin square design. Food allotments were offered to meet the individual maintenance energy requirement for each cat, estimated using historical colony data. Daily food intake, weekly BW and BCS were recorded throughout the study.^28^ On day 23 of each test period, cats were sedated using 0.3 mg/kg BW butorphanol (Zoetic, Kirkland, QC, Canada) and 0.005 mg/kg BW dexmedetomidine (Dexdomitor, Zoetis, Kirkland, QC, Canada) by IM injection for the placement of jugular catheters. An IV catheter was placed in the cephalic vein once cats were sedated to allow for IV propofol (1-4 mg/kg) induction (Fresenius Kabi Canada Ltd., Richmond Hill, ON, Canada). A maximum total dose of 8 mg/kg BW of propofol was administered to facilitate jugular catheter placement. After aseptic preparation of the area, the jugular catheter (MILA International Inc., Florence, KY, USA) was inserted and secured. Both the proximal and distal catheter lines were primed with heparinized saline solution (10 IU/mL) to maintain patency. Intramuscular administration of 0.02 mg/kg atipamezole (Zoetic, Kirkland, QC, Canada) was used to facilitate reversal. One fasted (07:50 h) blood sample of 3 mL whole blood and 6 post-prandial blood samples of 2 mL whole blood (1, 2, 3, 4, 5, and 6 h postprandial) were collected on day 24 of each period into BD Vacutainer Venous Blood Collection Tubes (serum tubes, Becton Dickson, Franklin Lakes, NJ, USA) and allowed to clot for isolation of serum insulin and lipoproteins. Samples were centrifuged (2500×*g* for 15 min at 4°C) and serum was separated using pipettes, aliquoted into Fisherbrand Microcentrifuge Tubes (Thermo Fisher Scientific, Rochester, NY, USA) and stored at −20°C until further analyses. Whole blood glucose concentration was measured immediately in duplicate at all time points using a portable glucose monitor (AlphaTRAK 2 Abbott Laboratories, North Chicago, IL) previously validated for use in cats.^35,36^

Serum insulin concentration was measured using the commercially available Mercodia Insulin ELISA (Mercodia AB, Uppsala, Sweden).^37^ The assay was performed following the manufacturer protocol and optical density was read at 450 nm (BioTek Absorbance Reader with Gen5, Agilent Technologies, Inc., Santa Clara, CA, USA). Standard curves were generated using BioTek Gen5 software (Agilent Technologies, Inc., Santa Clara, CA, USA). Samples were analyzed in duplicate, and the average concentration was used when the coefficients of variation (CV) were < 20%. If CV was > 20%, additional serum samples were run until a CV < 20% was achieved. Across the 8 ELISA kits, intra-assay reliability ranged from 6.6% to 8.2% and inter-assay reliability (*n* = 8) from the provided kit standards was 16.5%. The area under the curve (AUC) and incremental AUC (iAUC) for serum insulin (AUC_Ins_; iAUC_Ins_) and whole blood glucose (AUC_Glu_; iAUC_Glu_) concentrations over the 360-min post-prandial period were calculated using the trapezoidal method in SAS Studio (SAS Studio 3.8, SAS Institute, Cary, NC, USA) and the ratio of AUC_Ins_ to AUC_Glu_ and of iAUC_Ins_ to iAUC_Glu_ were calculated. Simplified measures of insulin sensitivity were calculated using fasted whole blood glucose and serum insulin concentrations for the insulin ($\mu$U/mL) to glucose (mmol/mL) ratio (Ins:Glu) and the homeostasis model assessment (HOMA) using the following equation^38^:


$$HOMA=\frac{Insulin\ \left(\mu U/ mL\right)\times Glucose\ \left( mmol/L\right)}{22.5}$$

Serum from fasted cats was sent to the Animal Health Laboratory, University of Guelph, for analysis of cholesterol, nonesterified fatty acids (NEFAs), high-density lipoprotein cholesterol (HDL-c), and triacylglycerol (TAG) using a Roche Cobas 6000 c501 analyzer (Roche Diagnostics, Basel, Switzerland). Very low-density lipoprotein (VLDL) and low-density lipoprotein cholesterol (LDL-c) were calculated as follows^39^:


$$VLDL= TAG\div 2.2$$

$$LDLc= Cholesterol-\left( HDLc+ VLDL\right)$$

### Statistical analysis

Statistical analysis was carried out using SAS Studio 3.8 (SAS Institute, Cary, NC, USA). Data are presented as least squared mean (SD) with significance set as *P* < .05. Normality of the residuals was assessed using the Shapiro–Wilk test and log transformation was used as needed to meet the assumptions of analysis of covariance (ANCOVA). Fasted measures (glucose, insulin, Ins:Glu, cholesterol, NEFA, HDL-c, TAG, VLDL, and LDL-c), AUC data (AUC_Ins_, AUC_Glu_, and AUC_Ins_:AUC_Glu_), and HOMA were analyzed using the proc GLIMMIX procedure with test diet and body condition as fixed effects, period as the random effect, and cat as subject. Post-prandial insulin and glucose concentrations were analyzed using the proc GLIMMIX procedure as a repeated measure of variance model with body condition and test diet as the fixed effect, time as the repeated measure, period as the random effect, and cat as the subject. A Tukey post-hoc adjustment was used to separate means when the fixed effect was significant using the covariance structure that resulted in the smallest Akaike information criterion value.

## Results

No adverse effects were observed throughout the study, and all 3 diets were well tolerated by the cats. One cat was removed because of behavior concerns from the lean group (*n* = 8) and another was removed because of BCS discrepancy in the obese group (*n* = 8).^28^ Daily energy intake was similar across all 3 diets (LP, 218.47, SD = 617.92 [*n* = 16]; LF, 220.42, SD = 623.44 [*n* = 16]; LC, 225.37, SD = 637.44 [*n* = 16] kcal/d) as previously described,^28^ but energy intake was higher for obese cats (255.05, SD = 721.39 kcal/d, *n* = 8) compared with lean cats (192.19, SD = 543.60 kcal/d, *n* = 8).^28^ For all cats, weekly BW and BCS remained consistent over the study period. Lean cats had a mean BW of 4.27 (SD = 12.08) kg and BCS of 4.8 (SD = 13.58). Obese cats had a mean BW of 5.82 (SD = 16.46) kg and BCS of 8.1 (SD = 22.91). The mean BW was not different between dietary treatment (*P*_Diet_ = .89) with mean BW for LP of 5.05 (SD = 14.28) kg, LF of 5.05 (SD = 14.28) kg, and LC at 5.04 (SD = 14.26) kg, and mean BCS was consistent across periods at 6.5 (SD = 18.38).

### Glucose

**Figure 1:**
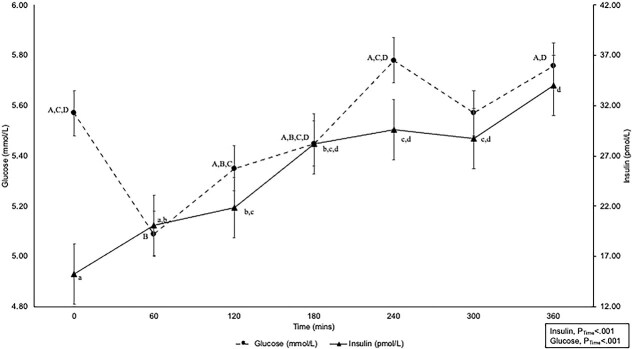
Pooled 360-min post-prandial whole blood glucose (mmol/L) and serum insulin (pmol/L) of lean and obese cats consuming either a low protein, low-fat, or low-carbohydrate diet for 4 weeks. Feeding occurred immediately after 0 min measurement. A, B, C, D denotes significant difference at each time point for whole blood glucose (mmol/L); a, b, c, d denotes significant difference at each time point for serum insulin (pmol/L).

After feeding, whole blood glucose concentrations decreased in all cats compared with fasted concentrations, and slowly increased thereafter, and returned to fasted concentrations by 240-min post-prandial and remained similarly to 360-min post-prandial concentrations (*P*_Time_ < .001) (Figure 1). Fasted whole blood glucose concentrations did not differ between lean and obese cats (*P*_BC_ = .50), among dietary treatments (*P*_Diet_ = .75), or for their interaction (*P*_BC^*^Diet_ = .71; Table 1). Similarly, no differences were found in post-prandial whole blood glucose concentrations between lean and obese cats at any time point (*P*_BC^*^Time_ = .67), among cats consuming the LP, LF, or LC diets (*P*_Diet^*^Time_ = .94), and no effect of their interaction was observed over time (*P*_BC^*^Diet^*^Time_ = .13; Table 1). The AUC_Glu_ and iAUC_Glu_ were similar among cats regardless of body condition and dietary treatment (*P*_BC_ = .41, *P*_Diet_ = .96, *P*_BC^*^Diet_ = .28, and *P*_BC_ = .66, *P*_Diet_ = .67, *P*_BC^*^Diet_ = .40, respectively, Table 1).

**Table 1:** Fasted and 360-min post-prandial whole blood glucose concentrations of lean (*n* = 8) and obese (*n* = 8) cats consuming a low-protein (LP, *n* = 16), low-fat (LF, *n* = 16), or low-carbohydrate (LC, *n* = 16) test diet for 4 weeks in a cross-over design.

	**Body condition**		**Test diet**			***P*-values**		
	**Lean**	**Obese**	**LP**	**LF**	**LC**	***P***_BC^*^Time_	***P***_Diet^*^Time_	***P***_BC^*^Diet^*^Time_
**Fasted glucose, mmol/L**	5.63 (0.37)	5.50 (0.40)	5.52 (0.45)	5.50 (0.48)	5.66 (0.45)	.50	.75	.71
**Post-prandial glucose, mmol/L**								
***60 min***	5.17 (0.40)	5.01 (0.40)	5.04 (0.37)	5.19 (0.40)	5.05 (0.37)	.67	.94	.13
***120 min***	5.41 (0.40)	5.29 (0.40)	5.36 (0.37)	5.41 (0.40)	5.28 (0.37)			
***180 min***	5.48 (0.40)	5.41 (0.40)	5.49 (0.37)	5.34 (0.40)	5.50 (0.37)			
***240 min***	5.57 (0.40)	5.59 (0.40)	5.47 (0.37)	5.70 (0.40)	5.56 (0.37)			
***300 min***	5.64 (0.40)	5.50 (0.40)	5.65 (0.37)	5.51 (0.40)	5.55 (0.37)			
***360 min***	5.93 (0.40)	5.59 (0.40)	5.79 (0.37)	5.74 (0.40)	5.75 (0.37)			
**AUC**_**Glu**_ **, mmol/L × min**	1 982.99 (104.96)	1 938.33 (106.43)	1 961.30 (90.71)	1 965.03 (92.77)	1 955.64 (90.74)	.41	.96	.28
**iAUC**_**Glu**_ **, mmol/L × min**	−14.37 (74.33)	−31.13 (76.03)	−19.42 (117.35)	4.88 (121.17)	−53.71 (117.30)	.66	.67	.40

Values expressed as LSM (SD).

Significance at *P* < .05.

Abbreviations: AUC = area under the curve; BC = body condition; iAUC = incremental area under the curve; LC = low carbohydrate; LF = low fat; LP = low protein; LSM = least square mean.

### Insulin

**Figure 2:**
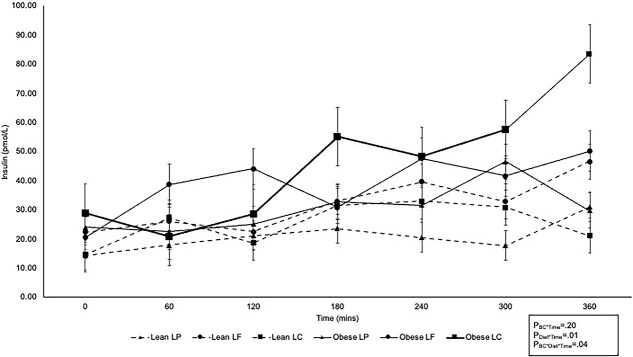
360-min post-prandial serum insulin curves for lean (*n* = 8; dotted lines) and obese (*n* = 8; solid lines) cats consuming an LP (*n* = 16; triangles), LF (*n* = 16; circles), or LC (*n* = 16; squares) diet for 4 weeks. Feeding occurred immediately after 0 min measurement. Abbreviations: LC = low carbohydrate; LF = low fat; LP = low protein.

Serum insulin concentrations increased over time for all cats regardless of body condition or diet (Figure 2). Similar fasted serum insulin concentrations were observed between lean and obese cats (*P*_BC_ = .35) and among diets (*P*_Diet_ = .63), and for their interaction (*P*_BC^*^Diet_ = .39; Table 2). Lean and obese cats did not differ in post-prandial serum insulin concentrations (*P*_BC^*^Time_ = .20); but cats consuming the LP diet had lower serum insulin concentrations compared with cats consuming the LF and LC diets at 240- and 360-min post-prandial (*P*_Diet^*^Time_ = .01; Figure 2). In addition, obese cats consuming the LC diet had higher serum insulin concentrations 240-min post-prandial compared with lean cats consuming the LP diet (*P*_Bc^*^Diet^*^Time_ = .04; Figure 2). At 360-min post-prandial, higher serum insulin concentrations were observed for obese cats consuming the LC diet compared with lean and obese cats consuming the LP diet and lean cats consuming the LC diet. Lean cats consuming the LF diet also had higher serum insulin concentrations at 360-min post-prandial compared with lean cats consuming the LC diet whereas obese cats consuming the LF diet had higher serum insulin concentrations at 360 min than obese cats consuming the LP diet (Figure 2). The AUC_Ins_ was not different between body condition and dietary treatments, or their interaction (*P*_BC_ = .09, *P*_Diet_ = .14, *P*_BC^*^Diet_ = .85; Table 2) but lean cats had a smaller iAUC_Ins_ compared with obese cats (*P*_BC_ = .04) whereas no differences were observed among dietary treatments or the interaction between body condition and diet (*P*_Diet_ = .35 and *P*_BC^*^Diet_ = .72, respectively; Table 2).

**Table 2:** Fasted serum insulin and insulin sensitivity parameters in lean (*n* = 8) and obese (*n* = 8) cats consuming a low-protein (LP, *n* = 16), low-fat (LF, *n* = 16), or low-carbohydrate (LC, *n* = 16) test diet for 4 weeks in a cross-over design.

	**Body condition**		**Test diet**			***P*-values**		
	**Lean**	**Obese**	**LP**	**LF**	**LC**	***P***_**BC**_	***P***_**Diet**_	***P***_BC^*^Diet_
**Fasted insulin, pmol/L**	12.81 (7.98)	17.21 (10.66)	13.21 (7.50)	15.67 (8.60)	15.80 (8.49)	.35	.63	.39
**Ins:Glu**	0.38 (.23)	0.54 (0.34)	0.40 (0.20)	0.48 (0.03)	0.48 (0.25)	.27	.59	.23
**HOMA**	0.57 (.45)	0.82 (0.65)	0.68 (0.54)	0.67 (0.51)	0.68 (0.51)	.36	.99	.54
**AUC**_**Ins**_ **, pmol/L × min**	9 556.81 (4 743.07)	13 825 (4 717.59)	9 579.05 (4 518.47)	12 691 (4 354.02)	12 803 (4 246.63)	.09	.14	.85
**iAUC**_**Ins**_ **, pmol/L × min**	3 075.89 (2 212.65)^A^	5 494.01 (2 149.07)^B^	3 136.35 (2 778.05)	4 731.37 (266.16)	4 987.12 (2 572.26)	.04	.35	.72
**AUC**_**Ins**_ **:AUC**_**Glu**_	4.66 (2.57)	7.26 (2.57)	6.51 (2.18)	6.67 (2.26)	4.72 (2.32)	.06	.05	.70
**iAUC**_**Ins**_ **:iAUC**_**Glu**_	31.88 (34.39)	5.17 (51.14)	18.18 (22.37)	4.14 (44.07)	92.56 (117.10)	.31	.17	.34

A, B denotes significant difference for BC along the row; a, b, c denotes significant differences for Diet along the row.

Values expressed as LSM (SD).

Significance at *P* < .05.

Abbreviations: AUC = area under the curve; BC = body condition; HOMA = homeostasis model assessment; iAUC = incremental area under the curve; Ins:Glu = fasted insulin to glucose ratio; LC = low carbohydrate; LF = low fat; LP = low protein; LSM = least square mean.

**Table 3:** Mean fasted serum concentrations of cholesterol, nonesterified fatty acids (NEFAs), triacylglycerol (TAG), and high-density lipoprotein cholesterol (HLD-c), low-density lipoprotein cholesterol (LDL-c), and very low-density lipoproteins (VLDL) of lean (*n* = 8) and overweight (*n* = 8) cats and of cats consuming a low-protein (LP, *n* = 16), low-fat (LF, *n* = 16), or low-carbohydrate (LC, *n* = 16) test diet for 4 weeks in a cross-over design.

	**Body condition**		**Test diet**			***P*-values**		
**(mmol/L)**	**Lean (*n* = 8)**	**Obese (*n* = 8)**	**LP (*n* = 16)**	**LF (*n* = 16)**	**LC (*n* = 16)**	***P***_**BC**_	***P***_**Diet**_	***P***_BC^*^Diet_
**Cholesterol**	6.61 (1.39)	7.11 (1.30)	7.17 (1.02)^a^	5.94 (1.02)^b^	7.47 (1.02)^a^	.48	<.001	.28
**NEFA**	0.68 (0.14)	0.76 (0.11)	0.70 (0.11)	0.69 (0.11)	0.77 (0.11)	.20	.25	.64
**TAG**	0.85 (0.25)	0.99 ± 0.08	0.83 (0.25)	0.89 (0.25)	1.04 (0.25)	.29	.14	.52
**HDL-c**	2.60 (.54)	2.80 (0.51)	2.69 (0.51)	2.62 (0.51)	2.80 (0.51)	.48	.71	.99
**LDL-c**	3.62 (1.33)	3.86 (1.24)	4.11 (0.99)^a^	2.92 (0.99)^b^	4.21 (0.99)^a^	.72	<.001	.49
**VLDL**	0.39 (0.11)	0.45 (0.11)	0.38 (0.11)	0.40 (0.11)	0.47 (0.11)	.28	.14	.52

A, B denotes significant difference for BC along the row; a, b, c denotes significant differences for Diet along the row.

Values expressed as LSM (SD).

Abbreviations: BC = body condition; HDL-c = high-density lipoprotein cholesterol; LC = low carbohydrate; LDL-c = low-density lipoprotein cholesterol; LF = low fat; LP = low protein; LSM = least square means; NEFA = nonesterified fatty acid; TAG = triacylglycerol; VLDL = very low-density lipoprotein.

No differences between lean and obese cats were observed for Ins:Glu or HOMA (*P*_BC_ = .27 and *P*_BC_ = .36, respectively) and both Ins:Glu and HOMA were similar among cats consuming the LP, LF, and LC diet (*P*_Diet_ = .59 and *P*_Diet_ = .63) regardless of body condition (*P*_BC^*^Diet_ = .23 and *P*_BC^*^Diet_ = .39; Table 2). The ratio of AUC_Ins_ to AUC_Glu_ was also similar among cats regardless of body condition (*P*_BC_ = .06), dietary treatment (*P*_Diet_ = .05), and their interaction (*P*_BC^*^Diet_ = .70). Similarly, no differences were found between lean and obese cats (*P*_BC_ = .31), cats consuming the LP, LF, or LC diet (*P*_Diet_ = .17), or the interaction of body condition and dietary treatment (*P*_BC^*^Diet_ = .38) for the iAUC_Ins_:iAUC_Glu_ (Table 2).

### Lipoprotein and lipids

Fasted serum concentrations of cholesterol, NEFA, TAG, HDL-c, LDL-c, and VLDL of lean and obese cats consuming a LP, LF, or LC diet are shown in Table 3. No differences were found between lean and obese cats for fasted serum cholesterol, NEFA, TAG, HDL-c, LDL-c, and VLDL concentrations (*P*_BC_ > .05). Cats consuming the LF diet, regardless of body condition, had lower fasting serum cholesterol (*P*_Diet_ < .001) and LDL-c (*P*_Diet_ < .001) concentrations compared with cats consuming the LP and LC diets. Diet did not affect fasting concentrations of NEFA, TAG, HDL-c, or VLDL in cats regardless of body condition (*P*_Diet_ > .05).

## Discussion

Contrary to previous reports,^23,40^ both lean and obese cats in our study had similar fasting and post-prandial whole blood glucose concentrations and no differences in AUC_Glu_ regardless of whether they consumed the LP, LF, or LC diet for 4 weeks. Of note, we identified that whole blood glucose concentrations decreased in the first 60-min post-prandial. This finding is consistent with those of previous studies in which peak whole blood glucose concentrations after a meal did not occur within the first 2 h post-prandial in contrast to other species such as dogs or humans.^41–44^ This result is likely because of the slower rate of glucose digestion and absorption in cats because of their lower amounts of pancreatic enzymes for carbohydrate digestion and their inability to upregulate glucose transporters in the intestinal brush border.^45–48^ However, obese cats responded differently to dietary interventions compared with lean cats regarding serum insulin concentrations. Furthermore, consumption of the LF diet in our study resulted in lower fasting serum cholesterol and LDL-c concentrations compared with the LP and LC diets as expected because of the lower fat intake.^49^ In contrast to previous reports,^50,51^ no effects of body condition on serum cholesterol, TAG, NEFA, and lipoprotein concentrations were identified.

Regarding diet effects, cats in our study did not exhibit differences in whole blood glucose concentrations when consuming the LP, LF, or LC diets, regardless of body condition. This finding supports the hypothesis that cats are metabolically flexible and can adjust the rates of carbohydrate oxidation and gluconeogenesis for the maintenance of blood glucose concentration.^23,24,27,52^ Our finding of no effect on fasted whole blood glucose concentrations in the present study aligns with results from a recent meta-analysis.^21^ However, the lack of differences in post-prandial concentrations of glucose might contradict previous observations. When carbohydrates were included at amounts exceeding most commercial extruded dry foods (NFE, 46%-52%ME), peak and mean post-prandial glucose concentrations were increased compared with LC diets (NFE, 19%-27%ME).^40,53,54^ Despite the higher post-prandial glucose concentrations, insulin concentrations were not different among diets.^40,53,54^ A previous study, however, observed no differences in glucose tolerance using IVGTT in healthy adult cats when fed a LC diet (NFE, 7%ME) compared to diets with carbohydrate content within a typical range for commercial cat food (NFE, 29%ME and NFE, 25%ME).^22^ In that study, the LP diet resulted in lower serum insulin concentrations compared with the LF and LC diets, suggesting that cats consuming the LP diet had higher insulin sensitivity.^22^ Our study builds upon these findings and support the hypothesis that a LP diet results in decreased amino acid intakes, and thus, decreased stimulation of the pancreatic β-cells.^22,55,56^ Alternatively, in our study, less insulin secretion may have been required to maintain blood glucose concentrations in the cats consuming the LP diet. This possibility could be attributed to the lower efficiency of carbohydrates as a source of glucose when compared with protein. Indeed, protein intake was higher in cats on the LF and LC diets, which, in comparison to the LP diet, required more insulin secretion. When protein intake is higher, cats can adjust gluconeogenesis accordingly. It could be that higher insulin concentrations were observed in cats on the LC and LF diets because of higher glucose output. It is likely that a combination of a decrease in amino acids for pancreatic β-cell stimulation and lower efficiency of utilizing carbohydrates occurred in the cats. More research in this area is required to elucidate the interactions of dietary macronutrients on the regulation of insulin and glucose in cats.

In our study, the difference in carbohydrate content among the test diets is narrower than in previous studies.^22,56^ In addition, the source of carbohydrates may have affected the findings. Carbohydrate sources can differ in carbohydrate distributions such as simple carbohydrate content, complex carbohydrate content, and starch gelatinization, granule structure, and fiber content, as well as alter the digestibility, in cat foods.^41,57,58^ In a previous study, corn grits and beet pulp were the major carbohydrate sources^40^; the test diets in another study utilized corn starch.^22^ Our study relied on oat groats and pea starch for carbohydrate content. Previously, a corn-based diet resulted in higher fasting and incremental glucose concentrations than a pea-based diet in healthy adult cats, but starch digestibility was similar between diets.^41^ Potential explanations for these findings could be differences in amylose and amylopectin, as well as their respective ratios, in various starch sources and their effect on glucose metabolism.^59,60,61^ In addition, NFE is only an estimate of the carbohydrate content and does not separate simple and complex digestible carbohydrates. Furthermore, the NFE estimation also includes indigestible components such as soluble fiber^33,62^ and the crude fiber analysis is merely an estimation of insoluble fibers and does not include soluble fiber.^63^ Limited information is available on how carbohydrate composition may affect glucose responses in cats,^41,57,58^ and even less data are available on the carbohydrate distributions in cat foods in general.^64^ Therefore, future research should consider how these differences may impact findings.

Obese cats consuming the LC diet in our study had the highest serum concentrations of insulin compared with both lean and obese cats fed the LP diet at 360-min post-prandial. Interestingly, we previously found that lean cats on LP and obese cats on LC had higher serum iAUC for leptin compared with obese cats on LP.^28^ Leptin in humans is thought to inhibit insulin secretion, whereas insulin triggers leptin secretion.^65^ However, in obese states, higher leptin concentrations and leptin resistance are observed.^66–69^ When considering our findings, obese cats consuming the LC diet may have experienced decreased ability of leptin to effectively inhibit insulin secretion resulting in higher insulin concentrations at 360 min post-prandial, but leptin resistance was not confirmed in obese cats in the previous study.^28^ In addition, rodent models previously have demonstrated that peptide-YY (PYY) acts on the pancreas to stimulate insulin secretion.^70^ The higher insulin concentrations in cats consuming the LC diet at 240 and 360 min also coincides with the higher PYY concentrations previously observed 60 and 120 min after consumption of a LC diet.^28^ However, more research on the relationship of PYY and insulin and glucose homeostasis in cats is needed. Future research should explore species differences of the relationships among leptin, PYY, insulin, and glucose.

Although a body condition and diet interaction effect was observed in our study, the lack of differences between lean and obese cats, regardless of diet, was unexpected. In humans, obesity is associated with higher circulating concentrations of free fatty acids (FFAs), TAG, cholesterol, VLDL, and LDL-c, and lower circulating HDL-c concentrations^71–73^ because of adipocyte hyperplasia and hypertrophy, leading to increased FFA flux to the liver and TAG accumulation, followed by enhanced VLDL synthesis and secretion^74,75^ and impaired lipoprotein lipase activity.^76^ Rather, exchange of cholesterol-esters from HDL-c to LDL-c and to VLDL occurs via cholesterol-ester-transfer-protein.^72^ In humans, this observation explains the lower HDL-c and higher VLDL and LDL-c concentrations observed with obesity.^72^ Similar dyslipidemia patterns occur in obese cats,^50,51^ but obese cats often exhibit higher circulating HDL-c concentrations compared with lean cats,^50^ particularly in short-term obesity, but long-term obese cats exhibited a similar pattern to humans for HDL-c.^51^ Duration of obesity appears critical, influencing both lipoprotein composition and TAG concentrations. In newly obese cats, the TAG in VLDL fractions were 180% higher compared with those during lean and long-term obese conditions, and were 500% higher compared with those in lean cats.^50,51^ The higher TAG in VLDL fractions also corresponded to higher plasma TAG concentrations in the long-term obese cats.^51^ Interestingly, in our study, although obese cats had higher fasting serum cholesterol, TAG, NEFA, VLDL, LDL-c, and HDL-c concentrations than lean cats, these differences were not significant. As we previously reported,^28^ results from dual-energy X-ray absorptiometry scans showed that the obese cats (BCS, 8 or 9 out of 9) had a mean body fat percentage of 26.2 (±1.3)% and 15.0 (±1.3)% for the lean cats (BCS, 4 or 5 out of 9). The corresponding body fat percentage to BCS observed previously has been reported.^77^ However, the obese cats had fluctuating BCS before the start of the study because of the previous studies in which they had been enrolled.^78–80^ Obese male cats in a previous study had a mean body fat percentage of 38.2 (± 5.0)%.^50^ Thus, the propensity and duration of the obese condition may be crucial to dyslipidemia in cats. However, information about changes in the density of lipoproteins may occur before circulating concentrations of lipoproteins and could provide additional insight into the mechanisms of the development of dyslipidemia in cats. Our study was limited in that density of lipoproteins was not measured. In addition, we utilized the Friedewald formula to calculate VLDL and LDL-c, which previously has been applied to cats,^39,79,81–84^ but has not yet been validated against laboratory measures such as electrophoresis.

In addition, few differences were noted between lean and obese cats regarding fasted and post-prandial whole blood glucose and serum insulin concentrations in our study. Previously, higher post-prandial glucose and insulin concentrations were observed in overweight and obese cats compared with lean cats.^85,86^ It has been estimated that each additional kg of excess BW decreases the insulin sensitivity of a cat by up to 30%.^23^ In humans, post-prandial hyperglycemia is described as concentrations > 7.7 mmol/mL^4^ and is thought to be similar in cats.^6,7,58^ However, in our study, lean and obese cats maintained post-prandial glucose concentrations below 7.7 mmol/L. Previously, we found no differences in serum leptin concentrations between lean and obese cats.^28^ Higher fasted leptin concentrations have a strong positive relationship to IR in cats and is postulated as a predictor for IR.^66^ The lack of difference is hypothesized to be a consequence of obesity severity and duration, which should be further investigated in cats to better understand the pathophysiology of obesity, leptin resistance, and IR. Furthermore, a previous study observed that obese cats had less expression of insulin sensitive transporters and insulin signaling genes.^10^ This scenario results in decreased glucose clearance and, subsequently, hyperglycemia.^9,87^ Chronic hyperglycemia causes exhaustion of pancreatic β-cells, ultimately resulting in decreased IR and glucose intolerance. In our study, the use of hyperglycemic clamp or IVGTT to assess insulin sensitivity and glucose tolerance was not available, but the use of fasted Ins:Glu and HOMA previously have been suggested as a simplified method to determine insulin sensitivity in cats.^38,88^ No differences in these measures were observed between lean and obese cats in our study.

Overall, our findings support previous research and emphasize the metabolic flexibility of cats, as obligate carnivores.^22,40,49,89^ Contradicting hypotheses that decreasing NFE content will decrease post-prandial glucose and insulin concentrations in cats and thereby aid in the prevention of IR onset in healthy or obese cats,^18,40,86^ we did not find that feeding the LC diet resulted in decreased post-prandial glucose or insulin concentrations in healthy lean or in obese cats. Rather, our results suggest that modulating protein, and consequently amino acid intake, may be more important in post-prandial insulin responses in domestic cats. However, more research is required to understand how the carbohydrate source and type affect glucose tolerance and insulin sensitivity in cats under healthy, obese, or diabetic conditions. Furthermore, cats in our study consumed one meal per day (08:00 h), but lower post-prandial insulin concentrations were observed in lean cats fed 4 meals daily.^90^ The role of feeding frequency, as well as diet and body condition, should be further explored. In addition, the duration and severity of obesity are likely to play important roles in IR, lipoproteins, and responses to dietary interventions and warrant further investigation.

## Supplementary Material

aalag013_Supplementary_Table_1

## Off-label antimicrobial declaration

The authors declare no off-label use of antimicrobials.

## Institutional animal care and use committee or other approval declaration

All experimental procedures were conducted in accordance with national and institutional guidelines for the care and use of animals in research and were approved by the University of Guelph Animal Care Committee (AUP #4865).

## Abbreviations

AUC: area under the curve
BCS: body condition score
BW: body weight
DM: type II diabetes
HDL: high-density lipoprotein cholesterol
HOMA: homeostasis model assessment
iAUC: incremental area under the curve
IR: insulin resistance
IVGTT: intravenous glucose tolerance test
LC: low-carbohydrate
LF: low-fat
LDL-c: low-density lipoprotein cholesterol
LP: low-protein
ME: metabolizable energy
NEFA: nonesterified fatty acids
NFE: nitrogen-free extract
TAG: triacylglycerol
VLDL: very-low density lipoprotein
