# Supplementary material for: Circulating lipid profiles and post-prandial glucose and insulin in response to dietary macronutrient composition in lean and obese cats
Source: J Vet Intern Med. 2026 Feb 17;40(1):aalag013. doi: 10.1093/jvimsj/aalag013 (PMC12910623; doi:10.1093/jvimsj/aalag013)
Supplement: aalag013_Supplementary_Table_1 [file aalag013_supplementary_table_1.docx]

**Supplementary Table 1.** Ingredient composition and proximate analysis (% as fed) of a low-protein (LP), low-fat (LF), and low-carbohydrate (LC) test diets formulated for adult maintenance using an isoenergetic approach with varying levels of the same ingredients fed to cats (n=18) for 4 weeks in a 3x3 Latin square design when offered to maintain body weight.

| **Parameter** | **LP** | **LF** | **LC** |
| --- | --- | --- | --- |
| **Ingredients (%)** |  |  |  |
| *Chicken meal* | 17.6 | 18 | 18.4 |
| *Fresh chicken* | 20.0 | 5.0 | 8.0 |
| *Dried chicken* | 1.1 | 17.1 | 14.5 |
| *Pea starch* | 19.8 | 17.8 | 6.8 |
| *Pea fibre* | 10.9 | 12.0 | 11.9 |
| *Egg powder* | 6.0 | 2.0 | 8.0 |
| *Poultry hydrolysate* | .5 | 7.7 | 8.0 |
| *Oat groats* | 7.5 | 10.6 | 9.7 |
| *Chicken fat* | 6.8 | 2.2 | 6.0 |
| *Fish oil* | 2.1 | .7 | 1.9 |
| *Dry palatant* | 1.3 | 1.3 | 1.3 |
| *Liquid palatant* | 3.0 | 3.0 | 3.0 |
| *Vitamin/mineral premix* | 2.7 | 1.7 | 1.7 |
| *Kelp* | .3 | .5 | .4 |
| *Salt* | .4 | .4 | .4 |
| **Proximate Analysis (% as fed)** |  |  |  |
| *Moisture​* | 8.25 ​ | 5.22​ | 6.05​ |
| *Dry Matter​* | 91.75 ​ | 94.78 ​ | 93.95 ​ |
| *Crude Protein​* | 31.24 ​ | 42.48 | 40.94 |
| *Crude Fat​* | 18.4 | 12.9 | 19.10 |
| *Crude Fibre​* | 4.40 | 4.80 | 4.70 |
| *Ash​* | 6.30 | 7.40 | 7.20 |
| *Total Dietary Fibre​* | 11.20 | 11.80 | 12.40 |
| *NFE^1^​* | 31.41 | 27.20 | 22.01 |
| *Metabolizable Energy^2^ (kcal/kg)​* | 3,756.75 | 3,535.30 | 3,826.75 |
| **Macronutrient Distribution (% ME)** |  |  |  |
| *Protein* | 28.0 | 40.0 | 36.0 |
| *Fat* | 40.0 | 30.0 | 41.0 |
| *NFE* | 32.0 | 30.0 | 23.0 |

LC, low-carbohydrate; LF, low-fat; LP, low protein; ME, metabolizable energy; NFE, nitrogen-free extract

^1^Calculated as: Nitrogen-Free Extract=100-(Crude Protein+Crude Fat+Crude Fiber+Ash) ^33^

^2^Calculated as: ME=10*(Crude Protein*3.5)+(Crude Fat*8.5)+(NFE*3.5) ^33^
